# Qualitative analysis of cocaine and heroin users’ main partner sex-risk behavior: is safety in love safety in health?

**DOI:** 10.1186/1940-0640-8-10

**Published:** 2013-04-23

**Authors:** Edward Bernstein, Valerie Ng, Lois McCloskey, Kathy Vazquez, Desiree Ashong, Stephanie Stapleton, Jasmin Cromwell, Judith Bernstein

**Affiliations:** 1 Boston University School of Medicine, Boston, MA, USA; 2 Boston University School of Public Health, Boston, MA, USA; 3 Department of Emergency Medicine, Boston University Schools of Medicine and Public Health, 1 Boston Medical Center Place, Boston, MA 02118, USA; 4 Beth Israel Deaconess Medical Center, 330 Brookline Avenue, Brookline, MA 02215, USA

**Keywords:** HIV, Main partner, Heterosexual transmission, Sexual profiling, Heroin, Cocaine, Intravenous drug use (IDU)

## Abstract

**Background:**

In 2009, 27% of the 48,100 estimated new cases of HIV were attributed to heterosexual contact with an infected or at-risk person. Sexually active adults are less likely to use condoms in relationships with main partners than with non-regular partners, despite general knowledge that condom use reduces HIV transmission.

**Methods:**

The purpose of this secondary qualitative analysis was to explore and contextualize perceptions of main partnerships, HIV risk, and attitudes toward condom use within main partner relationships among a subsample of intervention-arm cocaine- and/or heroin-using patients enrolled in a negative trial of brief motivational intervention to reduce the incidence of sexually transmitted disease and unsafe sexual behaviors. The open-ended portion of these interview audiotapes consisted of questions about perceptions of risk and attitudes about condom use with main partners. Enrollees were aged 18-54, English or Spanish speaking, and included in this analysis only if they reported having a main partner. We identified codes and elaborated important themes through a standard inductive three step coding process, using *HyperRESEARCH*™ software.

**Results:**

Among 48 interviewees, 65% were male, half were non-Hispanic white, over 60% were 20-39 years of age, 58% had intravenous drug use (IDU), and 8% were HIV-positive. Participants defined respect, support, trust, and shared child-rearing responsibility as the most valued components of main partner relationships. Condom use was viewed occasionally as a positive means of showing respect with main partners but more often as a sign of disrespect and a barrier to intimacy and affection. Enrollees appraised their partners’ HIV risk in terms of perceptions of physical health, cleanliness, and sexual and HIV testing history. They based decisions regarding condom use mainly on perceived faithfulness, length of involvement, availability of condoms, and pregnancy desirability.

**Conclusions:**

Risk appraisal was commonly based on appearance and subjective factors, and condom use with main sexual partners was described most often as a demonstration of lack of trust and intimacy.

**Trial registration:**

NCT01379599

## Background

Despite the decline of 9% in HIV transmission rates in the US from 2006 to 2009, the Center for Disease Control and Prevention (CDC) estimates that 1,148,200 people age 13 years and older were living with HIV in 2009, including 207,600 (18%) whose infection was undiagnosed [1]. Among new cases, 27% were attributed to heterosexual contact with a person with known HIV infection or high risk for infection [2]. Even among those with injection drug use (IDU), high-risk sexual behavior has been recognized as a more significant source of transmission than IDU [3-5].

Condom use is a recognized strategy for effectively reducing HIV transmission by 80% [6,7]. Nevertheless, sexually active adults are known to be more resistant to using condoms in ongoing relationships with their main partners than with non-regular partners [8-11]. Likewise, consistency of condom use is known to be higher with new and casual partners than with regular partners [12,13] and is inversely proportional to relationship duration [14]. Trends are similar among people with IDU. For example, among young adults with IDU, men with multiple partners were less likely to use condoms with their main partner [15].

From 2004-2009, we conducted a trial of motivational interviewing to encourage adoption of safe sex behaviors among emergency department (ED) patients whose use of heroin and cocaine resulted in a score of >3 on the Drug Abuse Severity Test (DAST). While the group as a whole (control and intervention groups, n=1030) showed a significant decline in unsafe sex with casual and transactional partners over time, there was no change at the 12 months in the rate of unsafe sex with main partners [16]. The brief motivational interview had no long-term impact compared with the control condition of assessment and referral for drug treatment only; this finding is supported by a meta-analysis of 37 randomized controlled intervention trials (88% of them limited to people with IDU) that reported only a transient impact on unsafe sexual practices [17].

We were intrigued by the intervention trial finding of resistance to change with main partners in a drug-using population and could find no literature comparing safe sex practices in people with cocaine and heroin use to attitudes and behaviors in the general population. For this reason, we undertook a secondary analysis of the interview portion of audiotapes originally recorded for purposes of intervention fidelity, limiting our analysis to tapes from participants who described having a main partner. Our approach was guided by the theory of reasoned action and planned behavior [18,19], which suggests that a person's behavior is determined by intention, which is a function of attitudes, norms, and perceived behavioral control. The purpose of this qualitative study was to explore and contextualize the perceptions of people with cocaine and heroin use regarding HIV risk and condom use within main partner relationships to see if we could identify specific characteristics of the expression of intention among those with IDU and enrich our understanding of determinants of resistance to change within main relationships.

## Methods

### Human subjects

The research protocol, including consent for interviews and taping, was approved by the Boston University Medical Center Institutional Review Board, and a Certificate of Confidentiality was issued by National Institute of Drug Abuse (NIDA). Enrollees received $10 in the form of a cash voucher for enrollment in the study.

### Scope of secondary analysis

The original intervention consisted of a semi-structured interview with two components: an inquiry about condom practices and views on condom use in different situations (pros and cons, benefits and limitations) followed by a motivational component focused on generating a menu of options for change and committing to change. We analyzed the descriptive component of these taped interviews for the purpose of this study, which was to generate information about perceptions of risk and attitudes about condom use with main partners among people with active drug use.

### Sample selection

Participants were selected from the 1030 people with cocaine and heroin use enrolled in a large randomized controlled trial to test the efficacy of a brief motivational intervention to increase safe sex behaviors and reduce the rate of sexually transmitted infections (STIs) and HIV among ED patients [16]. The study was conducted from November 2004 through May 2008 in the adult ED at Boston Medical Center, a US urban, academic level-I trauma hospital. Subjects eligible for the trial were aged 18-54, spoke English or Spanish, had DAST scores of >3 (representing moderate or severe drug use) [20], and agreed to HIV/STI voluntary brief counseling and testing. The control group was not interviewed; among the 513 in the intervention group, 173 consented to audio-recording of their interviews, and 138 tapes were of sufficient audio quality to allow for >80% transcription and coding. We selected 60 tapes at random for coding, stopping the selection process when we reached saturation of the codes. The current study focuses on information provided by the 48 enrollees in the randomly selected group of 60 recordings who reported being in a relationship with a main partner (defined as “people you have a relationship with, or commitment to, or people with whom you have sex regularly”).

### Data coding and analysis

We used established inductive coding methods and criteria to characterize important themes shared by all groups and to identify differences in the relative importance of these themes in subgroups. The analysis of the transcribed text involved three steps, which together constitute an iterative and inductive process of content analysis. In step one, researchers read through the interview transcripts and underlined important words or phrases. In step two, we derived meaningful codes from these words and attached the codes to segments of text. In step three, we identified overarching themes and labeled the text accordingly. We used *HyperRESEARCH*™ software [21], a text retrieval program geared toward in-depth exploration of data. Procedures are described in more detail in a similar study [22]. Study investigators trained and supervised a team of five research assistants. The research team met weekly for a month to discuss and code interviews together, address problems, and refine the coding system.

Differences in interpretation were discussed to reach consensus. Initial word identification codes were then compressed into second-level concept codes, resulting in a list of codes and sub-codes with standardized definitions. Prior to coding of all narratives, a subsample of 10 interviews were coded independently by each of the research assistants, and results for each of the 10 were compared across coders for inter-rater reliability (95% concordance). Definitions were then clarified and/or refined for items with lack of agreement. Using the *HyperRESEARCH*™ software, we generated reports for each major concept. Three consensus themes emerged from these reports, each focused on an aspect of main partner relationships: 1) how main partners are defined and valued; 2) how familiarity, trust, and respect between main partners affect condom use; and 3) how main partners appraise risk and adjust their sexual behaviors among main partners.

## Results

### Sample composition

Sixty-five percent of the 48 interviewees were men; 48% were non-Hispanic white, 42% were non-Hispanic black, and 10% were Hispanic. Sixty-two percent were between the ages of 20-39 years. Drug-use characteristics were as follows: 27% reported using only heroin, 31% reported using only cocaine, and 42% reported using both. Fifty percent reported IDU, and 8% were HIV positive (Table 1).

**Table 1:** Main partner characteristics: comparison between qualitative and quantitative study groups

**Variables**	**Qualitative** **(n=48)**	**Quantitative** **(n=1030)**
**Gender**		
Male	65%	67%
Female	35%	33%
**Race/ethnicity**		
Hispanic	10%	19%
Black	42%	39%
White	48%	41%
**Age (years)**		
20-29	31%	Mean, 35.8
30-39	31%	
40-49	33%	
50-59	4%	
**Drugs used (past 30 days)**		
Heroin	27%	20%
Cocaine	31%	27%
Both	42%	53%
**Injection drug use (past 30 days)**	58%	50%
**HIV-positive**	8%	8.8%

### Thematic material

We analyzed narratives for key themes that describe determinants of unsafe sex behaviors among men and women who use cocaine and heroin. We use exemplary quotes within each theme to convey these key ideas. For each, we note age; gender (M=male, F=female); race (W=white, Bl=black, H=Hispanic); drug of choice (C=cocaine, H=heroin, B=both); and HIV and IDU status.

#### Defining main partnerships

Main partner relationships are consistently defined and valued based on duration, mutual support, and respect. There was a general consensus that main partner relationships are defined primarily by length of time in the relationship, a sense of mutual responsibility, financial support of children in common, respect, and support. To a lesser extent, they are bounded by exclusivity.

Reflecting a common viewpoint, one male respondent stated, “*I’m only with one woman and hopefully if things work out, I’ll only be with that woman for a long time.*” (24, M, W, B, IDU)

Highlighting the commonly held belief that mutual support and friendship form the most important ingredient of main partnerships, one respondent explained, “*I made some true friends. I don’t have too many—you know, my main partner is my best friend. That's the people I count on today.*” (40, M, H, B)

Another noted how mutual support is associated with her drug use: *“[My husband]’s a big support when he knows that I’m doing it [drugs]. For months, he had no idea so that’s a dishonesty between us (giggles nervously); me being totally dishonest with him. But when I’m honest with him and he knows I’m having a hard time, he’s very supportive and I can always talk to him.”* (31, F, H, B)

Main partner relationships were often defined by sharing children in common and taking responsibility for them. As one male respondent put it, “*I do the home thing—the daddy thing and everything else—cook, clean, and whatever—and she loves that. She doesn’t have to do anything.”* (42, M, Bl, C).

In some instances, men described their firm financial and material support of their children as payback for their partners’ loyal support through years of tough times brought on by drug use. One respondent elaborated, “*I have made a change as far as my financial responsibility, at least to my baby daughter. My checks, they go directly to her and her mother, and if I need something, . . . I put this woman through so much, and she’s still there. . . . At least financially, you know, right now, she has that coming from me . . . we are talking and working at being back together.*” (44, M, Bl, B)

#### How familiarity, trust, and respect with main partners affect condom use

A few respondents noted that the use of condoms can be a way to show respect: *“Well, I’ll sit my girlfriend down like I always do and we talk and tell her that I found out I got hepatitis B. And that I can’t—we can’t continue to have sex without protection.”* (37, M, Bl, B)

“*It [using a condom] was her wishes, you know. We just respected each other. I think it was the thing to do. That’s what she wanted to do*.” (42, M, Bl, C)

As relationships progressed and time passed, many participants reported that there was no point in using condoms: *“We were going to have sex, and she’s like you don’t have a condom on you. I go, no, I don’t. So, she wouldn’t have sex with me. And, the next time we went to have sex, we had just sex without the condom, when the condom broke. So we said f--- it, and had sex or whatever. . . . And, then you know, I, it seems like for me when I’m with a partner, after a little bit, you know what I mean, it’s, there’s no point of a condom .*” (26, M, W, B, IDU)

*“Because she’s my main partner and I—I trust her. I mean, I don’t think she has something and she—that she’s hiding from me. And I don’t have nothing that I’m hiding from her.*” (40, M, H, B)

For some, the desire to express love and feelings of safety decreased the desire to use condoms*.*

The lack of worry led one couple to stop using condoms: *“At first, we did use condoms, when me and him first started dating. We used condoms for about three months and eventually, we just fell in love so quickly that we just—we didn’t want to use them. We just—I wasn’t worried about anything, he wasn’t worried about anything, and we just felt safe together.”* (20, F, W, B, IDU)

Women also described not using condoms in long-standing relationships, whether or not they are monogamous: *“With my main partner, we’ve been together for two and a half years, and he’s only had sex with . . . I know every single person that he’s had sex with, and I’m not really worried about him.”* (20, F, W, B)

Women were more likely to describe not using condoms in romantic terms: *“It just felt so right not to, you know what I’m saying? It was just one of those things like I love him and he loves me.”* (26, F, H, B)

*“We used condoms for about three months and eventually we just fell in love so quickly that we just—we didn’t want to use them. We just—I wasn’t worried about anything, he wasn’t worried about anything, and we just felt safe together.”* (30, F, W, B)

#### How main partners appraise risk and adjust their behaviors

Both men and women appraise their partners’ risk and adjust their sexual behaviors differently within main and other partnerships. This appraisal of risk is based on a broad spectrum of assumptions, some true and others not. Participants’ appraisal of risk within their main partnerships revolved largely around knowledge of a partner’s sexual history and prior testing as well as the experience of testing together. They tended to use (or not use) condoms based on perceived monogamy, length of involvement, the desire to have or avoid a pregnancy, as well as the availability of condoms. A majority of respondents had misconceptions about risk assessment, and these misconceptions played a large part in making decisions about condom use. For example, several interviewees interpreted physical cleanliness and good health as evidence of low risk for HIV and STIs. Most important, many respondents expressed the belief that what they valued most—trust and respect—were protective against HIV and STIs. They assumed that if they showed trust by refraining from condom use, the partner would behave in a way to validate that trust. Thus, they perceived condom use as a reflection of distrust or assumed immorality of the partner. Several examples illustrate how these notions play out, often in overlapping ways, as main partners assess risk and make choices about condom use.

The partner’s level of hygiene was thought to be a predictor of HIV/STI risk: “*With someone I know who washed their body every day—taking care of their health—as far as my wife, I don’t mind not using a condom. I feel safe. I know I’m safe with her.”* (48, M, W, C)

Participants thought that knowledge of their partner’s sexual history, such as the number or names of prior sexual partners, decreased their susceptibility to any risk, providing them a false sense of security. Condoms were used until main partner status (test results or sexual history) was known. Many stated that getting tested together assured them that they were both clean: *“I just don’t like them. I’m not gonna. I know we are both clean. We have both been tested together. So I don’t see any need to use them.*” (23, F, Bl, B, IDU)

Many participants used length of time as a determinant of exclusivity and, ultimately, of really knowing their partners. The perception of risk was diminished when participants were with someone for some length of time and when believed to be in a perceived monogamous relationship: *“There is nothing I don’t like about condoms. I should rephrase that. There is nothing wrong with them. If I didn’t have one partner for the last seven years or was sleeping around, I would absolutely, definitely use them.”* (34, F, W, B, IDU)

*“I would be totally shocked if one of them* [tests] *came back positive. I would be thrown for a loop, because all this time I’ve been monogamous and expecting that nothing would harm me like that, you know what I mean?”* (24, F, W, C)

On the other hand there were also those who voiced a need for condom use with their main partners when they felt unsafe, wanted to avoid pregnancy, were concerned about the risks they were taking themselves, or wanted to protect their partners from sequelae of their own risky behavior: *“If I felt that my main partner is cheating, then yes, of course I would use a condom.” (40, M, H, B)*

*Well, mainly to prevent pregnancy. I have three. This will be my fourth. I’m fighting for custody. I’m trying to get them back. And I don’t want to just keep having, especially with me being an addict. If I can’t take care of them—they don’t deserve it. So I want it to be planned.”* (31, F, Bl, H)

“*Well, now because I know how important it is, you know what I'm saying, when I'm out there tricking and doing what I need to do to get my drugs, that I need to have some kind of protection, because it's a lot out there. You know? And, I have a significant other and I don't want to bring him home anything*.” (36, F, Bl, B)

Others agreed with the connection between their reason for selecting their partner (high morality) and her risk-free status, choosing not to use a condom for this reason: *“If I have chosen to be in a relationship with this woman, and I believe that this person is a decent, moral person, I don’t need to wear a condom because, God knows, wouldn’t have ever chosen a partner that had an STD.”* (50, M, W, C)

In a more practical vein, some respondents reported not using condoms because they disrupted intimacy. Condoms were seen as an unnecessary physical barrier to flesh-to-flesh contact: “*It is about affection. So you know, I don’t think it is real affectionate having a piece of rubber between you*.”(32, M, B, H, IDU)

Use of a condom in a developing relationship can be misunderstood as lack of trust when partners are not forthcoming about risks related to drug use or their own positive HIV status: *“We got into this big thing where she thought I was wearing a condom to protect myself from her. And I didn’t want to divulge my own information to her. And that seems to be one of the main things. If you’re not telling somebody why you’re wearing the condom, then they take it personally that it’s got to do with them. They can’t accept the fact and leave it alone.*” (43, M, W, B, IDU, HIV)

## Discussion and conclusions

Several qualitative studies have focused on African Americans [23-27], as their rate of new HIV diagnosis has been increasing faster than other racial or ethnic groups [28]. Other studies have focused on HIV-positive individuals [29,30]. One qualitative study specifically examined the effect of cocaine and heroin on sexual performance and pleasure [31]. The description of themes identified in this study is one of the first to examine the perceptions and behaviors that influence safe sex practices in main relationships among a diverse group of cocaine and heroin-using men and women.

It is no surprise that our interviewees were resistant to condom use with main partners; they resembled people without drug use in that respect. In a recent study of a national probability sample, only 14% of men and 13% of women reported that they used condoms with a main partner at last penile-vaginal intercourse (PVI), compared with 25% of men and 31% of women who used condoms at last PVI with casual partners [32]. In our parent study among a diverse sample of 1030 drug-using ED patients, at baseline, only 17% of enrollees reported using condoms with their main partner at last PVI compared with 47% use with casual partners [33]. This study highlights the intricate relationship between condom use and common notions of protection afforded by duration of relationship and perceptions of trust and faithfulness in that relationship.

Drug use, with its increased risk of sexually transmitted disease, appears to represent an added layer of complexity in the choices people make about sexual relations and condom use, but the issues presented here have been documented in the general public as well. For example, personal cleanliness was cited in an earlier qualitative study as a factor in the decision-making process for low-income African Americans [27].

Respect, support, trust, honesty, common child-rearing, and mutual responsibility were described by participants as valued components of a main partner relationship. Cocaine and heroin use are commonly thought to isolate people from social supports and family ties [34], yet these analyses suggest that people with drug use place the same importance on the core values of trust and faithfulness within main partnerships as do others. In fact, these values appear to lie at the heart of their choices about condom use or nonuse, playing a powerful role along with risk appraisal based on concrete knowledge of their partners’ prior sexual history and recent testing status. Interviewees based their risk appraisal on knowledge of their partner’s sexual and testing history, and made condom choices based on perceived monogamy, length of involvement, pregnancy desires, and availability of condoms. At the same time, participants wanted to believe that faithfulness, moral decency, and physical cleanliness were protective against STIs or HIV, ideas that are not supported by evidence. Several respondents described the tension between secrecy and transparency about drug use, but everyone who discussed this issue shared a desire to maintain intimacy while high or addicted.

Men and women did not appear to differ in the importance they assigned to maintaining trust and intimacy within main partner relationships. Results from gender-stratified analyses have been mixed, with some studies citing the influence of perception of power on condom decision-making for women [35], and others finding an association between perceptions of power and condom use that is not related to gender [36].

Interestingly, men and women in our sample described factors that inform their appraisal of risk within their main partnership in much the same way as has been reported for others with high-risk status. Syringe-sharing behavior among people with IDU provides a case in point. Among participants in a recent study of IDU in Russia [37], there was a significant association between high levels of trust and the likelihood of risky injection practices. Those who were very trustful had a false sense of security in assuming that syringe-sharing partners in same network would reveal whether they were HIV positive; and, like the participants in our study, they were also concerned that questioning their partners would threaten or strain important relationships. The association between vulnerability to HIV and social coding of intimacy in modern societies may explain why main partners might put themselves at high risk in their attempts to maintain relationships. Martin Blas explains it this way: “If we want to make a relationship last, we must take the chance to trust. Unfortunately, what is safe for love can be dangerous for health, and what is safe for health may pose a threat to love. Sexual risk-taking thereby represents an attempt to stabilize the intimate relationship in order to insure its duration” [38]. Based on our study findings, two questions arise for further investigation: To what extent do persons who use drugs resemble the general population in their attitudes toward condom use with main partners, despite their high-risk status? And, if the public health goal is for safety in love to be congruent with safety in health, how do we change the basis for risk appraisal of main partners in this high-risk group to be more in line with science and evidence?

This study had several limitations. The source for this secondary analysis was transcriptions of audiotapes of interviews conducted as the initial conversational component of an intervention, and there were no control group tapes available for comparison. Generalizability from qualitative analysis is inherently limited. However, the interviewees in this study were drawn from the largest “out of treatment” sample of people with heroin and cocaine use reported at a single urban medical setting. Because the intervention group and the control groups were similar in demographic and drug use characteristics at baseline and did not differ in adoption of safe sex practices at the 12 month follow-up, it is reasonable to make the assumption that attitudes and behaviors reported by the intervention group are equally likely to have surfaced in the control group if interviews had been conducted with them at baseline.

In this study, we did not have a sufficient sample to conduct stratified analyses of differences in perceptions and behaviors by IDU status or type of drug used. Therefore, reported themes should be considered a starting point for further investigation of the role of trust and intimacy in condom use decisions for individuals whose drug use puts them at high risk for HIV and STI transmission. Despite these limitations, study results suggest that, among people with heroin and cocaine use, with and without IDU, both subjective perceptions of risk and beliefs about the importance trust in main partner relationships play an important role in resistance to condom use with main partners.

## Competing interests

The authors declared they have no competing interests.

## Authors’ contributions

EB is the corresponding author and principal investigator who designed and conducted study, trained coders, interpreted data, lead the writing and editing, and gave final approval of the version to be published. VG coded interviews, analyzed themes, conducted the literature search, and participated in writing and editing of the manuscript. LM participated in the interpretation of the interview data findings, conceptualizing and writing the results section. She reviewed and offered substantive critiques and revisions to manuscript drafts throughout the process. KV took prime responsibility in coordinating the coding process, coding the case interviews used for the study, and extracting themes and direct quotes to use in the manuscript. She took prime responsibility for the first draft of the results/key findings, the methods section, and the demographic table included in the manuscript. She provided comments and revisions to the manuscript. DA was the study coordinator who contributed to the conception and design, acquisition of data, and analysis and interpretation of data. She was involved in drafting and revising the manuscript. SS coded interviews and participated in the analysis of themes, reviewed literature, and commented and suggested revisions to manuscript. JC coded interviews and participated in the analysis of themes and commented on the manuscript. JB, co-principal investigator, designed and conducted study, trained coders, interpreted data, co-led the writing and editing, and gave final approval of the version to be published. All authors read and approved the final manuscript.
